# Characterisation of Zamorano-Leonese Donkey Milk as an Alternative Sustainably Produced Protein Food

**DOI:** 10.3389/fnut.2022.872409

**Published:** 2022-04-08

**Authors:** Irene Albertos, María López, José-María Jiménez, María José Cao, Alfredo Corell, María José Castro-Alija

**Affiliations:** ^1^Universidad Católica de Ávila (UCAV), Ávila, Spain; ^2^Recognized Research Group, University of Valladolid, Valladolid, Spain; ^3^Faculty of Nursing, University of Valladolid, Valladolid, Spain; ^4^Department of Immunology, University of Valladolid, Valladolid, Spain

**Keywords:** donkey milk, Zamorano-Leonese breed, protein, functional food, minerals, vitamins, sustainability

## Abstract

The Zamorano-Leonese donkey is the local breed of the Castilla y León region of Spain and is a protected endangered species. The best way to preserve it is to explore viable alternatives such as milk production. Unlike other donkey breeds, this one has not been previously characterised. The aim of this work is the complete nutritional characterisation of its milk for human consumption, either directly or as an ingredient, to meet the new consumer expectations of sustainability and health concerns. This breed did not differ from others in terms of amino acid and protein profile. Its low concentration of β-lactoglobulin may be correlated to a low allergenicity. The presence of lactozyme and lactoferrin, which are potent antimicrobials, stand out among the proteins. This milk presented a higher content of unsaturated fatty acids, being oleic fatty acid the main one. Zamorano-Leonese donkey milk did have a higher content of vitamin C, riboflavin, folic acid and vitamin E than the other donkey breeds. It also had a high concentration of vitamin D despite its low-fat content. However, its mineral concentration was lower than other donkey breeds in line with its lower ash content. In terms of micronutrients, it had a high amount of zinc and selenium. Based on these results we can conclude that donkey milk is a food and/or ingredient with beneficial effects on cardiovascular health and the proper functioning of the immune system, as well as being a good source of protein. Therefore, donkey milk from this local species from Spain is a food and/or ingredient with beneficial nutritional properties and sustainable from an environmental point of view.

## Introduction

The sustainable production of food of animal origin is one of the greatest challenges faced by the food system. In this regard, the European Green Deal (1) encourages a model of extensive livestock farming based on a more sustainable, organic, circular economy and animal welfare production techniques. Spain has a high potential to develop this extensive livestock farming due to its climate and soil characteristics. Our country has 50.6 million hectares, out of which only 17.5 million hectares are arable. Despite the high potential Spain has for its livestock and pasture resources, extensive livestock farming has been losing relevance in our country, decreasing from 23 million hectares in the 1980s to 5.8 million hectares in 2020 (2).

Currently, most of the milk produced in Spain comes from cows, sheep, goats and buffalo, with bovine milk accounting for 7.251 million tonnes in 2020 (3). Donkey milk is currently the focus of considerable research attention because of its similarity to human milk. It has also been studied for its potential benefits for human health due to its antioxidant and antimicrobial properties, modulation of gastrointestinal flora, stimulation of the immune system, reduction of the glycaemic index, etc. However, all studies have been conducted *in vitro* or on experimental animals, and clinical trials are needed for more scientific evidence (4). One of its most promising applications will be as an alternative to cow’s milk in cow’s milk protein allergies. This is very prevalent in infants in the first year of life in around 1.5–3%, which is when milk is the main food source of the diet (5). In this regard, donkey milk is more tolerated than other milks as a substitute for cow’s milk (6).

The Zamorano-Leonese is the local breed of the region of Castilla y León in Spain and is a protected endangered species. This breed belongs to the same family as other European donkeys (*Equus africanus asinus*). The donkey has a large body, a very voluminous head with wide and enlarged ear pinnae and a long and abundant fur. The presence of this donkey was first documented in the time of the Catholic Monarchs and had its heyday in the 19th and early 20th centuries. It has been traditionally used for agricultural work and domestic chores. Specifically, the area where the characteristics of this breed were shaped was the western region of the province of Zamora (54.6% of the specimens are concentrated in this province), which has a typically continental climate with hot, dry summers and cold winters. The farming of these animals is usually extensive or semi-extensive. In the latter case, they graze for a few hours a day and are then gathered indoors at night. Their diet is based on fodder produced on the farm and small amounts of concentrated feed (barley, rye and oats). In the event of a lack of pasture, some animals are kept in transhumance (7). This breed is currently used for agricultural work, short-distance transport and accompanying sheep. These tasks have fallen into disuse due to the introduction of agricultural machinery, and the population that used to rely on them is ageing. If all this is added to the fact that it is an animal with low fertility, this results in this breed of donkey being in danger of extinction. According to data from the Asociación Nacional de Criadores de Raza Asnal Zamorano-Leonesa (8), there are currently only 1,448 donkeys in Spain. It is this association that promotes the cultural and recreational importance of the Zamorano-Leonese as a domestic animal. Due to its good character, this breed has been used in animal-assisted therapy projects (8). The conservation of this donkey is of great importance to preserve biodiversity and also to contribute to economic development in areas heavily affected by depopulation. Milk production from these donkeys can be a viable alternative to maintain the species. Furthermore, this milk can be a new source of nutrients such as protein with low allergenicity. Unlike other donkey breeds in Italy, China, and the Balkan peninsula that have been extensively studied, milk from the Zamorano-Leonese donkey breed has not been previously assessed; there is no published work characterising this milk. This fact justifies the need to conduct this study, as breed is one of the factors that may significantly impact on the milk composition (3).

Therefore, this work presents a complete physico-chemical and nutritional characterisation of donkey milk from the Zamorano-Leonese donkey breed, comparing it with other donkey milk breeds and with other types of milk in order to understand its potential as a protein food or source of nutrients that can be isolated therefrom.

## Materials and Methods

### Materials

#### Reagents

The reagents were purchased from Panreac (Panreac Química, Barcelona, Spain) and Sigma (Sigma-Aldrich Chemical Co, Steinheim, Germany). All solvents were HPLC-LC grade (Lab-Scan, Dublin, Ireland).

#### Milk Collection

The milk was collected from the Zamorano-Leonese breed on a farm located in Sieteiglesias de Tormes (Salamanca). The animals (*n* = 8) were healthy primiparous or multiparous animals kept in extensive livestock farming and under organic production. The jennies were from 4 to 8 years old and had an average life weight of 270 ± 30 kg. During the first month and a half the milk was exclusively used for breeding. From that point onward, two litres a day were milked, saving approximately eight litres for the foal to cover its needs during this growth stage. All animals were fed on pasture, forage *ad libitum* (organic alfalfa) and oat supplementation for pregnant and lactating animals, and in general for all animals in winter.

The donkeys were milked by hand twice a day, about 4 h apart, yielding one litre per milking. The foal stayed with its mother 16 h a day and they were separated only a few hours before milking. Sampling was carried on September and October of 2021 when the jennies were between 60 and 120 days of lactation. Individual milk was collected every 2 weeks (4 sampling times). The milk was collected and transported under refrigerated conditions (4°C) to Villamayor (Salamanca). Pasteurisation was performed in a water-bath at 75°C for 15 s. The vitamin and mineral content between raw and pasteurised milk was compared; all analyses were performed in triplicate.

### Methods

#### Physico-Chemical Properties and Proximate Composition

The pH of the milk was measured at room temperature with a digital pH metre (pH-metre model 507, Crison, Barcelona, Spain). Milk acidity was measured by volumetric titration with sodium hydroxide (0.111 N) using 1% phenolphthalein in ethanol as titrant. The results are expressed in grams of lactic acid per litre of milk (gL^–1^).

The gross energy expressed in kJ/100 g is calculated by multiplying the nutritional composition by the coefficients. Moisture and ash were determined gravimetrically at 110 and 530°C, respectively. Fat was determined gravimetrically by extraction of fat in an alcohol-ammonia solution following the Röse-Gottlieb method (9). Milk protein was measured using the Kjeldahl method (10). Carbohydrates were quantified by difference. The results are expressed as a percentage (g in 100 g of product). The lactose monohydrate content was determined according to the official AOAC method 984.15 (11).

#### Fatty Acid Profile

An analysis of the fatty acid composition of the fat extract of the milk sample extracted by the Röse-Gottlieb method (9) was carried out. The lipid phase was dissolved in 1 mL hexane and mixed with 100 μL of 0.5 M methanolic KOH for 10 min at room temperature. The top layer was transferred to a 2 mL vial. Analysis of fatty acid methyl esters (FAMEs) was carried out by gas chromatography using an Agilent 7890 (Agilent Technologies, Palo Alto, CA, United States) equipped with a 60 mm × 0.32 mm DB-23 column (0.25 μm thick) and a flame ionisation detector. The carrier gas was helium. The oven temperature was set at 50°C for the first 7 min and increased to 200°C at a rate of 25°C per min. Subsequently, it was increased to 230°C at a rate of 3°C per min and maintained for 26 min. The injector and detector temperatures were 250 and 280°C, respectively. Hexane extract (1 μL) was injected in split mode (25:1) and the FAMEs were identified by comparison of the retention times of the standard (mixture of 37 FAMEs, Supelco, Sigma-Aldrich). The percentages of saturated, monounsaturated and polyunsaturated fatty acids were calculated, and *trans* fatty acids were determined. The polyunsaturated/saturated ratio (PUFA/SFA), the omega-6 to omega-3 ratio (n6/n3 ratio) and the atherogenic index (AI) [C12:0 + (4 × C14:0) + C16:0]/?UFA were also determined.

#### Amino Acid Profile

The milk was centrifuged, removing the upper fat layer, and hydrolysed using 6 M HCl. For tryptophan determination alkaline hydrolysis was performed (4 M NaOH). The hydrolysate was centrifuged and the supernatant was used for amino acid profiling after filtration with a 0.22 μm syringe filter. The analyses were performed on a liquid chromatograph with Agilent 1200 HPLC-FLD fluorescence detector (Agilent Technologies, Palo Alto, CA, United States). There were two phases, one phase prepared with 140 mM sodium acetate and 17 mM triethylamine adjusted pH to 4.95 with phosphoric acid. Eluent B was acetonitrile diluted in water. The flow rate was 1.0 mL/min and was measured at a length of 248 nm.

#### Protein Fraction Characterisation

The defatted milk sample was dissolved in buffer (50 mM Tris–HCl pH 6.8, 2% SDS, 10% glycerol, 1% β-mercaptoethanol, 12.5 mM EDTA and 0.02% bromophenol blue). Samples were heated at 95°C for 5 min and the supernatant was used in electrophoresis. Polyacrylamide gel electrophoresis (PAGE) was carried out in a vertical electrophoresis apparatus (Protean II, Bio-Rad, Richmond, CA, United States) at 75 V for 4 h in a cold chamber. The separation gel was stained with Coomassie blue for 12 h and destained for the same time. The gel image was densitogrammed using FluorChem software to quantify the percentage of each band with respect to the total proteins.

#### Vitamins

##### Water-Soluble Vitamins

Determination of water-soluble vitamins was performed by removing protein and fat with chloroform extraction and centrifugation.

The determination of vitamin C was performed by mixing the milk with dithiothreitol before the addition of metaphosphoric acid. The mixture is then allowed to react for 15 min so that all the dehydroascorbic acid is reduced to ascorbic acid. The sample is then filtered and measured on an Agilent 1200 liquid chromatograph (Agilent Technologies, Palo Alto, CA, United States) equipped with a diode-UV detector. A C18 reverse phase column (5 μm, 150 × 4.6 mm) (Teknokroma Analítica S.A., Barcelona, Spain) operated isocratically with two mobile phases consisting of Milli-Q water with acetic acid (0.1% v/v) and methanol at a ratio of 95:5 (v/v) was used. It was measured at a wavelength of 254 nm. Results were expressed as μgL^–1^.

Vitamins B_1_, B_2_, B_6_ and B_9_ were quantified on an Agilent 1200 liquid chromatograph (Agilent Technologies, Palo Alto, CA, United States) with Agilent 6500 Accurate Mass Spectrometer (Agilent Technologies, Palo Alto, CA, United States) for which the samples were previously filtered with nylon filters. The samples were evaporated with nitrogen gas at room temperature. The dried extract was reconstituted with 500 μL of mobile phase A [water containing 5% acetic acid, 0.2% heptafluorobutyric acid (HFBA) and 1% ascorbic acid]. These samples were vortexed for 10 min and filtered on ultrafree-MC units (PVDF 0.45 μM) at 12,000 g for 3 min at room temperature. The filtered extracts are directly transferred to the amber vial for injection. Results were expressed as μgL^–1^. Separation was done with a Waters BEH C18 reverse phase column (2.1 × 100 mm, 1.7 μm) (Waters, Barcelona, Spain). The mobile phase consisted of: 20 mM ammonium formate (phase A) and methanol (phase B) with a flow rate of 0.35 mL min^–1^. The gradient was as follows: phase A 99% for 0.5 min, 92% for 2 min, 10% for 2.5 min and held for 1 min before returning to initial conditions and rebalancing for 2 min (12). Results were expressed as μg/L.

Vitamin B_12_ was quantified microbiologically according to the AOAC standard (13). The results were expressed as μg/kg.

##### Fat-Soluble Vitamins

For the determination of fat-soluble vitamins (vitamin A, D_3_ and E) it was necessary a saponification according to the official CEN methods (14–16).

Vitamin A and D_3_ were quantified as follows: 40 μL were injected into an Agilent 1200 liquid chromatograph (Agilent Technologies, Palo Alto, CA, United States) equipped with a diode-UV detector. A Nucleosil 100 silica column (250 mm × 4.6 mm) (Macherey-Nagel, Düren, Germany) with a flow rate of 1.45 ml min^–1^ was used with a mobile phase of hexane/dioxane/2-propanol (96.7: 3: 0.3 v/v/v). This chromatograph was linearly connected to an Agilent 6500 Accurate Mass Spectrometer (Agilent Technologies, Palo Alto, CA, United States). A fragmentation voltage of 200 V, a drying gas flow rate of 5 L min^–1^, a nebuliser pressure of 60 psig and a drying gas and vaporiser temperature of 350°C were used (17).

Vitamin E was determined by the official AOCS method (18) using an Agilent 1200 liquid chromatograph (Agilent Technologies, Palo Alto, CA, United States) equipped with a diode detector. The extracted fat sample was analysed by the Rose-Gottlieb method (9) and tocopherol acetate used as an internal standard. A 20 μL aliquot was injected into a normal phase column (250 mm × 4.6/mm, 5 μm) (Teknokroma Analítica S.A, Barcelona, Spain) at 30°C. An isocratic phase of hexane and 2-propanol in a 99.6:0.4 (v/v) ratio, with a flow rate of 1.2 mL min^–1^ was used. Detection was performed at a wavelength of 292 nm for tocopherol and 284 nm for tocopherol acetate. Results were expressed as μg/L.

#### Minerals

The minerals were determined by inductively coupled plasma mass spectrometry (ICP-MS) 7850 ICP-MS Agilent (Agilent Technologies, Palo Alto, CA, United States). The sample was previously incinerated in a muffle at 530°C for 10 h, then treated with hydrochloric acid and filtered. The accuracy of the analytical procedure was assessed by analysis of reference skimmed milk powder.

### Statistical Analysis

Descriptive results were presented as means ± standard deviation. Statistical analysis were performed by Statgraphics Centurion XVI. The effect of stage of lactation on the investigated variables was not significant and not further considered in this paper. Comparison of statistical significance due to pasteurization were analysed by Student’s t-distribution for paired samples at 95% of significance level, after the demonstration of normality using the Shapiro-Wilk test.

## Results

### Physico-Chemical Properties and Proximate Composition

The pH of the donkey milk was 7.16 ± 0.1 and its acidity was 0.035 ± 0.002 g of lactic acid in 100 g of milk.

In terms of proximate composition, donkey milk provides 155 kJ in 100 g, with water as the major component (90.95 ± 0.42%). The ash, protein and lipid contents were 0.36 ± 0.03, 0.56 ± 0.02, and 1.68 ± 0.06%, respectively. The lactose content was estimated at 6.6 ± 0.1%.

### Fatty Acid Profile

Table 1 shows the fatty acid profile of Zamorano-Leonese donkey milk. The most abundant saturated fatty acid is palmitic (21.56 ± 1.15), followed by capric (5.68 ± 0.05), lauric (5.20 ± 0.06), myristic (4.73 ± 0.02), caprylic (3.23 ± 0.05) and stearic (1.15 ± 0.00). Linolenic acid (14.31 ± 0.07) and alpha-linolenic acid (12.32 ± 0.04) are the most predominant within the omega-3 (ω3) and omega-6 (ω6) series, respectively (Table 1).

**TABLE 1:** Fatty acid profile.

Butyric C4:0	0.11 ± 0.00
Caproic C6:0	0.31 ± 0.01
Caprylic C8:0	3.23 ± 0.05
Capric C10:0	5.68 ± 0.05
Undecylenic C11:0	1.14 ± 0.01
Lauric C12:00	5.20 ± 0.06
Tridecylic C13:00	0.14 ± 0.00
Myristic C14:00	4.73 ± 0.02
Pentadecylic C15:00	0.31 ± 0.00
Palmitic C16:00	21.56 ± 1.15
Heptadecanoic C17:00	0.30 ± 0.00
Stearic C18:00	1.15 ± 0.00
Myristoleic C14:1	0.26 ± 0.00
Palmitoleic C16:1	3.18 ± 0.01
Heptadecenoic C17:1	0.41 ± 0.00
Oleic C18:1n9c	25.12 ± 1.15
Linoleic C18:2n6 (c9, c12)	14.31 ± 0.07
Linolenic C18:3n3 (c9, c12, c15)	12.32 ± 0.04
Dihomo-γ-linolenic C20:3n6	0.29 ± 0.00

*Mean values ± standard deviation, n = 12.*

Considering the distribution of the fatty acid profile, unsaturated fatty acids (UFA) (55.09 ± 0.31%) are the most abundant, followed by saturated fatty acids (SFA) (43.91 ± 0.25%). Monounsaturated fatty acids (MUFA) (29.03 ± 0.16%) are present in a higher concentration than polyunsaturated fatty acids (PUFA) (27.06 ± 0.15%). In this latter, the content of omega 3 (ω3) and omega 6 (ω6) was 12.32 ± 0.07% and 14.74 ± 0.08%, respectively.

### Amino Acid Profile

The most prevalent amino acids were glutamic acid, proline, aspartic acid, leucine, lysine and valine. The concentration of essential amino acids represents 54% of the total amino acids in Zamorano-Leonese donkey milk. The amino acid profile of the milk analysed is shown in Table 2.

**TABLE 2:** Amino acid profile of Zamorano-Leonese milk.

Essential	
Histidine	0.02 ± 0.00
Leucine	0.15 ± 0.01
Lysine	0.13 ± 0.08
Phenylalanine	0.09 ± 0.05
Valine	0.15 ± 0.07
Tryptophan	0.01 ± 0.00
Threonine	0.05 ± 0.01
Methionine	0.03 ± 0.01
Isoleucine	0.12 ± 0.07
**Non-essential**	
Arginine	0.08 ± 0.01
Aspartic acid	0.16 ± 0.02
Alanine	0.06 ± 0.00
Tyrosine	0.06 ± 0.00
Proline	0.16 ± 0.06
Glycine	0.01 ± 0.00
Serine	0.08 ± 0.01
Glutamic acid	0.35 ± 0.10
Cysteine	0.00 ± 0.00

*Results expressed in%. Mean values ± standard deviation, n = 12.*

### Protein Profile

**FIGURE 1:**
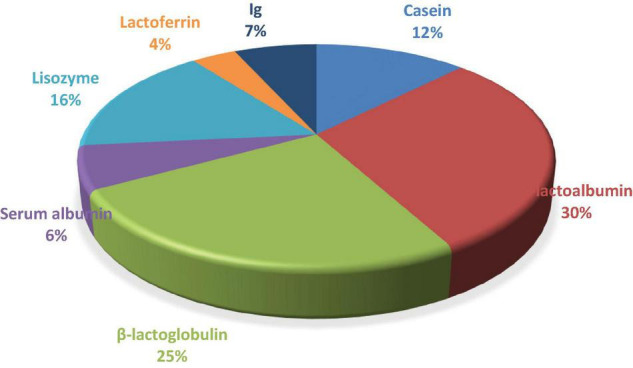
Distribution of donkey milk proteins analysed by polyacrylamide electrophoresis expressed as a percentage with respect to total proteins (*n* = 12).

The protein profile was quantified as a percentage and calculated from the gel (Figure 1). Caseins and whey proteins represent the major part of the donkey milk protein fraction. The main components of donkey milk from a quantitative standpoint were β-lactoglobulin, α-lactoglobulin, casein and lysozyme. Other more minor components detected were immunoglobulins and lactoferrin (Figure 1).

### Vitamins

In terms of water-soluble vitamins, the Zamorano-Leonese donkey milk showed high levels of vitamin C (63 ± 0.3 mg L^–1^). As for group B vitamins, the values are shown in Table 3. This milk was particularly notable for its high folic acid content. These water-soluble vitamins were not affected by the pasteurisation process, where there were no significant losses compared to untreated donkey milk (data not shown).

**TABLE 3:** Water-soluble B vitamins content of fresh milk from Zamorano-Leonese donkey.

	B Vitamins content
Thiamine (B_1_) (μL/L) Riboflavin (B_2_) (μL/L) Pyridoxine (B_6_) (μL/L) Folic Acid (B_9_) (μL/L) Cyanocobalamin (B_12_) (μL/L)	200 ± 0.10 345 ± 0.22 200 ± 0.13 525.8 ± 1.3 3.69 ± 0.01

*Mean values ± standard deviation, n = 12.*

**FIGURE 2:**
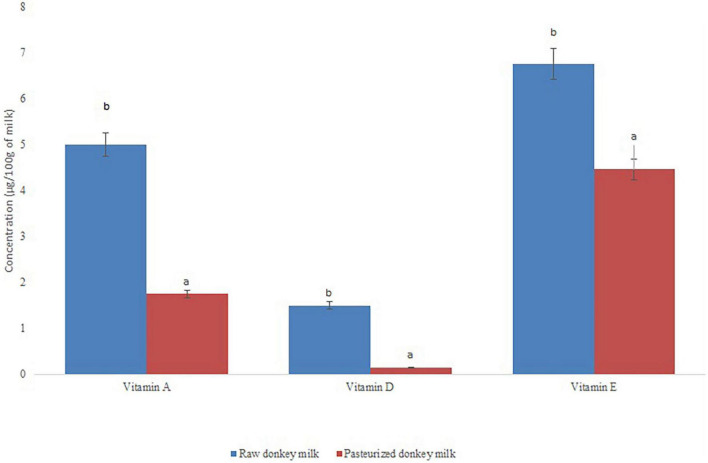
Concentration of fat-soluble vitamins in raw and pasteurised milk from the Zamorano-Leonese breed. Different letters mean significant differences between samples (*p* < 0.05).

The results for fat-soluble vitamins decrease significantly with heat treatment as can be observed in Figure 2. This is particularly significant in the case of vitamin E with a reduction of 66.22%, while for vitamin A and D it is 35 and 10%, respectively.

### Minerals

The concentration of minerals in Zamorano-Leonese donkey milk is shown in Table 4. There were no significant differences between heat treated milk and raw milk (data not shown).

**TABLE 4:** Mineral content of Zamorano-Leonese donkey fresh milk.

	Mineral content
Calcium (mg/kg) Phosphorous (mg/kg) Sodium (mg/kg) Magnesium (mg/kg) Potassium (mg/kg) Zinc (μg/kg) Iron (μg/kg) Copper (μg/kg) Selenium (μg/kg)	676 ± 0.70 518 ± 0.53 173 ± 0.40 84 ± 05 863 ± 1.53 2184.73 ± 20.81 100 ± 0.77 150.9 ± 2.5 107.1 ± 7.5

*Mean values ± standard deviation, n = 12.*

The most prevalent minerals were potassium, calcium and phosphorus. Zinc was the main micronutrient in this milk.

## Discussion

### Physico-Chemical Properties and Proximate Composition

The physico-chemical properties of donkey milk analysed were pH and acidity. The pH values obtained in our work were very similar to those of Addo et al. (19) at 7.19 and Salimei et al. (20) at 7.18. In other breeds such as dwarf Indian grey, the pH in donkey milk ranges from 7.10 to 7.28 (21). In a review conducted by Altomonte et al. (22) the pH value of donkey milk ranges between 7.2 and 7.5. This pH is similar to that of human and mare’s milk. Donkey milk has a more alkaline pH compared to milk from other widely consumed species such as cow, sheep and goat (23, 24). These higher pH values can be attributed to a lower casein and phosphate content (25). On the other hand, the acidity value found in our work was slightly lower compared to other study where it ranged between 0.049 and 0.054 (22).

The proximate composition of the donkey milk obtained in our study was compared to other donkey breeds and to milks of different species. This latter comparison is shown in Table 5.

**TABLE 5:** Proximate composition of our sample of Zamorano-Leonese donkey milk compared to other milk species.

	Donkey	Human (22)	Cow (33)	Goat (34, 36)	Sheep (36)	Camel (35)	Mare (37)
Moisture (%)	90.95 ± 0.42	90.5	87.5	87.8	88	86,8	NF
Ash (%)	0.36 ± 0.03	0.2	0.76	0.8	0.9	0.9	0.4
Fat (%)	0.56 ± 0.02	3.1	3.7	3.5	7.9	4	1.2
Protein (%)	1.68 ± 0.06	1.2	3.2	3.4	6.2	3.5	2.1

The determined energy content of Zamorano-Leonese donkey milk was in line with those found in other studies on donkey milk (from 313 to 414 Kcal/L). The caloric content was similar to that found in skimmed cow’s milk. The difference in energy value compared to breast milk is noteworthy, despite the fact that they are similar in other respects. Specifically, human milk has 400 Kcal/L, while cow’s milk contains between 620 and 650 Kcal/L (26).

Moisture content was similar to that obtained in other donkey breeds (20, 21, 27). Moisture content is very constant in donkey milk and does not depend on factors such as breed, day of lactation, year of lactation or number of milkings (20). Donkey milk is characterised by a higher water content compared to cow’s milk (87.5%) and human milk (90.5%) (Table 5).

Zamorano-Leonese donkey milk presented an ash content of 0.36%. Other dwarf Indian donkey breeds showed values of 0.40% (21), while Martina Franca and Ragusana breeds had an average of 0.39% (20), although these values varied depending on the lactation period. In the review by Altomonte et al. (22) the ash values of donkey milk varied between 0.3 and 0.4%. Ash values obtained in this study matched exactly the ash content of the Aminata breed from central Italy. In the work by Guo et al. (28) Chinese donkeys of the Jiangye breed was characterised and it was concluded that donkey breed does not affect the ash content. However, the lactation period does have an impact: the ash content decreases during lactation. The highest ash content is found in the first month of lactation when the foal is fed exclusively on milk and therefore the mineral requirements are greater for growth. Comparing the ash content with other species (Table 5) shows that it is very similar to that of mare’s milk (29). Donkey milk has more minerals than human milk, whose content is between 0.17 and 0.2% (22). However, its content is very low compared to cow’s milk, which is twice as high (Table 5).

The lipid content of this breed is low compared to the dwarf Indian grey which contains between 0.7 and 0.8% fat (21). The fat content of the Zamorano-Leonese breed is more similar to geographically closer species such as the autochthonous donkey from Greece and Cyprus, which contained 0.52% (30). The Martina Franca breed from Italy presented very similar values to the ones found in this study with an average of 0.54% (31). The Amiata breed from central Italy also had a similar fat content, namely 0.53% (27). However, the fat content was lower (0.38%) in the study by Salimei et al. (20); this work was carried out on Martina Franca and Ragusana breeds also in Italy. Fat composition, in contrast to ash, can be affected by the lactation period. There is a significant fat loss from the first month of lactation to 210 days (31). In this study, this is not relevant, as during the first month of lactation the donkey’s milk is used exclusively for breeding.

The fat globules are very small, half the size compared to other milks such as ruminants’ ones. This has an impact not only on technological aspects but also on its digestibility. Donkey milk may be more easily digestible due to the larger contact surface for lipase action (32).

The fat content of donkey milk is lower than in human milk (3.1%) (Table 5) and is equivalent to skimmed cow’s milk. It also has the advantage of preserving all fat-soluble nutrients. Other milks have a much higher fat content, such as cow’s milk (3.7%) (33), goat’s milk (3.5%) (34), camel’s milk (4%) (35) or sheep’s milk (7.9%) (36). Even mare’s milk, while similar in other aspects of its nutritional composition, differs substantially in fat content (1.2%) (37) (Table 5).

The protein concentration of Zamorano-Leonese donkey milk was slightly higher than that found in milk from the Nordestina breed from Brazil with 1.50% (38). It was also higher than in the autochthonous donkey from Greece and Cyprus, which only contained 1.22% (30). Similar values were found in the Martina Franca and Ragusana breeds in Spain with 1.61% (19) and in Italy with 1.72% (20). The Aminata breed in Italy also presents a similar protein percentage (1.63%). Higher values were found in other geographically more distant breeds such as in India with a content varying between 1.78–1.96% (21) and 1.5–1.8% in China (28).

Table 5 shows that donkey milk has a higher protein content than human milk (22) but much lower than other milks (33–36); it is about half that of cow’s milk. The differences are especially significant in the case of sheep’s milk and it also presents lower levels than mare’s milk (37).

The lactose content of this breed was similar to that found in other studies (12, 13, 20, 22, 30). Therefore, the lactose content is independent of the breed as reported in the work of Salimei et al. (20). This content is higher than that of cow’s milk (4.4–4.9%) (33) and similar to the one found in human milk (6.3–7%) (22). This makes it palatable and favours fermentation, contributing to the increased bioavailability of calcium. Lactose in human milk is the main energy source for the newborn. In addition, it is often bound to oligosaccharides, which are resistant to digestion and stimulate the growth of bifidobacteria in the colon. These microorganisms can prevent the growth of pathogens (39).

### Fatty Acid Profile

Zamorano-Leonese donkey milk has a lower concentration of saturated fatty acids and a higher concentration of polyunsaturated fatty acids than other donkey breeds (27, 31, 38). This may be related to their diet, in contrast to ruminants, where fatty acid modifications occur with biohydrogenation reactions in unsaturated fatty acids. In monogastric animals, these reactions do not occur and therefore the fatty acid profile of the milk will be dependent on their diet. In the work of Valle et al. (40), the composition of milk was determined by diet, with other factors such as breed and stage of lactation playing a negligible role. The donkeys in this study were fed exclusively on forage. In the study by Valle et al. (40), it was observed that animals fed on forage had a higher concentration of linolenic acid. This is because this fatty acid is the main component of fresh grass. In the absence of biohydrogenation, there is a large transfer of this fatty acid to donkey milk (40) as occurs in this work. The higher concentration of polyunsaturated fatty acids may explain the partial inhibition of saturated fatty acid synthesis in the mammary gland (41, 42).

The fatty acid profile has health related implications and impact on the nutritional quality of the fat. The predominant fatty acid in this milk (oleic) is associated with a reduction in cardiovascular risk. The Zamorano-Leonese donkey has the highest levels of this fatty acid compared to other donkey breeds such as the Aminata (27), Martina Franca (31), and Nordestina (38).

Another widely used index is the ratio of polyunsaturated to saturated fatty acids (PUFA/SFA), which was 0.62 for this breed and it is related to cardiovascular health. PUFA can reduce low-density lipoprotein cholesterol (LDL-c) and plasma cholesterol, while SFA contribute to an increase in plasma cholesterol. If we compare this ratio (PUFA/SFA) in donkey milk with other breeds (Table 6), it can be observed that Zamorano-Leonese donkey milk has the highest ratio and therefore the most positive contribution to health.

**TABLE 6:** Distribution of the fatty acid profile and different nutritional indexes to assess the quality of the fatty acid profile in the milk of the Zamorano-Leonese donkey compared to other breeds.

	Zamorano-Leonese donkey	Aminata donkey (27)	Martina Franca donkey (31)	Nordestina donkey (38)
SFA (%)	43.91 ± 0.25	50.20	51.98	48.82
UFA (%) MUFA (%) PUFA (%)	55.09 ± 0.31 29.03 ± 0.16 27.06 ± 0.15	50.2 35.5 14.70	48.02 28.00 20.02	52.54 37.67 14.87
PUFA/SFA	0.62	0.29	0.39	0.30
ω6/ω3	1.16	–	1.81	1.19

*Mean values ± standard deviation, n = 12.*

A further advantage of its fat composition is the ω6/ω3 fatty acid ratio (1.16) is lower than in other breeds (Table 6). This may be related to a lower production of pro-inflammatory eicosanoids, which may favour protection against pathologies associated with metabolic syndrome such as diabetes or cardiovascular diseases. Besides its high concentration of medium-chain fatty acids (lauric acid and capric acid), with implications for vasodilatory activity and, together with short-chain fatty acids, in the body’s defence against oxidative stress (43, 44).

Donkey milk, despite its low-fat content, stands out for its richness in polyunsaturated fatty acids. In fact, this donkey milk provides more polyunsaturated fatty acids (0.15 g/100 g) than other milks with a much higher fat content, such as cow’s milk (0.12 g/100 g) and goat’s milk (0.09 g/100 g) (36, 37). Donkey milk is rich in polyunsaturated fatty acids, mainly ω-3 fatty acids [alpha-linolenic acid (ALA), docosahexaenoic acid (DHA), and eicosapentaenoic acid (EPA)], which may be involved in the neurophysical development of the newborn (43, 44).

The atherogenicity index (AI) was developed by Ulbricht and Southgate (43) as an improvement to the PUFA/SFA ratio. The AI includes the major saturated fatty acids (C12:0, C14:0, and C16:0) that are considered to be proatherogenic, which favour lipids to cells in the circulatory and immune systems. This index is calculated by dividing it by unsaturated fatty acids (UFA), which inhibit plaque accumulation by reducing levels of phospholipids, cholesterol and esterified fatty acids (44). Compared to other milks and dairy products, the donkey milk index (0.81) is lower than the values reported for these foods, which ranged from 1.42 to 5.13 (44).

### Amino Acid Profile

**FIGURE 3:**
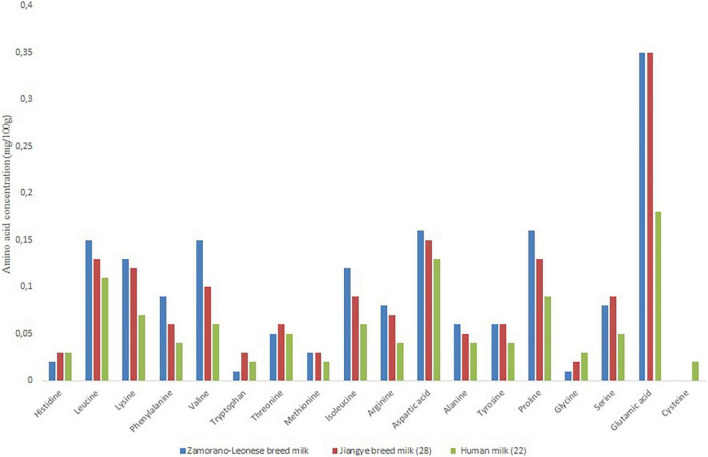
Amino acid concentration (mg/100 g of milk) of Zamorano-Leonese breed milk in comparison with Jiangye breed milk (28) and human milk (22).

The amino acid profile composition of the Zamorano-Leonese breed is similar to that found in other breeds from a qualitative point of view. Moreover, this composition is constant throughout the whole donkey’s lactation period (28). A quantitative comparison shows that the amount of amino acids in the Zamorano-Leonese donkey is very similar to that found in the Jiangye breed from China (28) (Figure 3). The results of this breed were higher than those of Nordestina from Brazil (38) and lower than those of the dwarf Indian grey breed (21).

Donkey milk is very rich in essential amino acids compared to other mammalian milks, second only to mare’s milk (14, 27, 37, 38). Another important characteristic is its similarity to mother’s milk, which will be relevant from a nutritional point of view. In fact, infant milks are assessed in accordance with their amino acid content (22).

### Protein Profile

The results obtained by SDS in the Zamorano-Leonese breed show a similar profile to that found in other donkey breeds such as Martina Franca (20), Ragusana (20), and Jiangye (28). However, it is certain that there are quantitative differences in the composition (20), such as a lower concentration of lactoferrin and lysozyme in the Zamorano-Leonese breed compared to Martina Franca and Ragusana (20).

Donkey milk contains a much higher α-lactalbumin content than human and cow’s milk (22, 37). This protein has well-recognised nutritional properties, especially in infant nutrition. It has a high tryptophan content, an essential amino acid that has been positively related to brain development in newborns. It also contributes to the functioning of the nervous system because it is a direct precursor of serotonin or niacin (vitamin B_3_) (45). In contrast, the concentration of β-lactoglobulin is lower than that found in cow’s milk (37). This protein is not present in human milk and is considered the major allergen in cow’s milk, in addition to caseins (40). In the protein fraction, the lysozyme content of donkey milk is much higher than that of human milk (22) and cow’s milk (where it is non-existent) (37). Lysozyme has an important antibacterial function with a role in the immune response (39). Lactoferrin, also associated with antimicrobial properties (46, 47), is much higher in donkey milk than in cow’s milk (37), whereas its concentration in human milk is not as high (22).

### Vitamins

#### Water-Soluble Vitamins

The vitamin C values found in the present study were higher than in others (57 mg/L) (48). The vitamin C content is slightly higher than in human milk (60 mg/L) and much higher than in cow’s milk (27 mg/L) (49). Overall, the vitamin C content of donkey milk in various studies has ranged from 12 to 57 mg/L, similar to human milk (38–53 mg/L) (22). Donkey milk is therefore a good source of vitamin C, with 500 mL of donkey milk covering the recommended daily intake (25 mg) in children aged 0–12 months (50). Vitamin C is remarkable for its antioxidant power, iron absorption contribution and, moreover, is involved in the production of collagen. It also prevents atopic dermatitis in high-risk children (22, 51). Vitamin C intake may also be important in situations such as ageing, pregnancy and lactation with increased requirements of 70, 80, and 100 mg/L compared to adults (60 mg/L).

The thiamine (vitamin B_1_) concentration was similar to the results found by Vicenzetti et al. (49). Thiamine values are similar to cow’s and mare’s milk (48) and much higher than human milk (49). However, the thiamine content of donkey milk varies considerably (22). Values ranging from the ones found in this work to 2,550 μg/L have been found in the published studies (22). These differences may be due to the fact that the studies were done in other donkey breeds, but even within the same breed there may be differences due to feeding, lactation period, season or climate conditions (52).

Zamorano-Leonese donkey milk showed riboflavin (vitamin B_2_) values above those described in other studies (49) but within the same range (30). These values are higher than the ones found in human milk, but lower than those found in the milk of other animals such as cows and goats (22, 49). The concentration of riboflavin in donkey milk would be similar to the found in mare’s milk (40). These values are in line with the riboflavin values found in donkey milk, which ranged from 40 to 970 μg/L in several studies (30). Riboflavin is very sensitive to light but very resistant to temperature (48).

This breed presented lower pyridoxine (vitamin B_6_) values than those found in other donkey milk studies (48, 49). These discrepancies in other species such as the mare have been found to be due to the lactation period (53). Donkey milk has much higher levels of vitamin B_6_ compared to human or mare’s milk but similar to cow’s or goat’s milk (48, 49). Vitamin B_6_ deficiency is common in the elderly and in women of childbearing age. This vitamin is involved in the immune response, in the proper functioning of the immune system and in metabolic pathways of amino acids, lipids and gluconeogenesis (48, 49).

Folic acid (vitamin B_9_) levels were slightly higher than those found in other studies (48, 49). The folic acid content is 3 and 59 times higher compared to human and cow’s milk, respectively (49). These proportions are even higher compared to other species such as mare’s or goat’s milk (49). This fact is very relevant in infant feeding, as milk is an important source of folic acid in children under 1 year of age (49). It is also important in pregnant women since folic acid is necessary for foetal development due to its participation in the synthesis of nucleic acids and cell division (54). This vitamin is significant in other groups such as the elderly because of its role in Alzheimer’s disease (55). This can be extended to the adult population in general, since in developed countries one of the most common deficiencies is megaloblastic anaemia. In addition, deficiency of this vitamin is also associated with the development of cardiovascular diseases and cancer (55).

In the present study, cyanocobalamin (vitamin B_12_) was detected in this donkey milk. Other authors have not detected this vitamin in donkey milk (48, 49). Vitamin B_12_ is produced in the intestine and its metabolism in donkeys has not been thoroughly investigated (48, 49).

Other studies indicate that the minimum cobalamin level in donkey milk is 1.1 μg/L, although no maximum limit is established (22). Donkey milk contains less cyanocobalamin than human milk (22) and these differences would be even greater if compared with other species such as cow or sheep (36). These differences between equines and ruminants could be due to their different digestive systems (49).

The levels of these water-soluble vitamins remained stable during pasteurisation. Claeys et al. (42) report that there is no loss of B vitamins even with more severe treatments such as conventional sterilisation. These authors argue, on the basis of research, that from a nutritional point of view the effect of thermal processing on vitamins is negligible. Other storage factors such as oxygen or the presence of light affect them much more than heating.

#### Fat-Soluble Vitamins

Overall, the fat-soluble vitamin content of donkey milk is lower than in other milks (cow, sheep or goat) due to its lower fat concentration (36).

The Zamorano-Leonese donkey milk presented differences in vitamin A content compared to other donkey breeds studied (21, 48, 56). The vitamin A concentration found in this study is lower than in human milk and much lower than in cow’s milk (22, 48, 56). It also differs from mare’s milk (57), whose composition is quite similar in all other nutrients.

Donkey milk has a very low vitamin E content compared to human milk and other species such as cow’s milk (30, 48, 56). The vitamin E content of Zamorano-Leonese donkey milk (6.75 ± 0.02 μg/100 mL) is higher than that found in other donkey breeds (21, 48).

In contrast, the concentration of vitamin D was higher in donkey milk than in cow’s milk (58) and human milk (59). This is significant, since vitamin D is present in few dietary sources and vitamin D deficiency is relatively common. Zamorano-Leonese donkey milk has slightly lower levels (1.5 ± 0.0 μg/100 mL) than those found in the literature in other breeds such as Amiata (60). The role of this nutrient in bone formation and the prevention of osteoporosis is well known (61) but it is also related to the immune system. In fact, the benefits of vitamin D in COVID-19 have been recently studied, with an inversely proportional relationship between the serum concentration of this vitamin and the severity of the disease (62).

This vitamin was the most stable to heat treatment, dropping from 1.5 ± 0.0 μg/100 mL in raw milk to 0.15 ± 0.0 μg/100 mL in pasteurised milk. Martini et al. (60) obtained similar results, showing that both raw and pasteurised donkey milk were a good source of this vitamin.

### Minerals

The calcium content of Zamorano-Leonese donkey milk was slightly lower than that found in the Nordestina (38) or Martina Franca (63) and much higher than in the dwarf Indian grey breed (21). Calcium levels in donkey milk are quite high; 1 litre of donkey milk covers 84.5% of the recommended dietary allowance (RDA). It contains twice as much calcium as human milk (22) and has a lower concentration than cow’s, sheep’s or goat’s milk (36). This can be attributed to the low amount of ash in donkey milk compared to other milks.

Phosphorus levels measured in Zamorano-Leonese donkey milk were lower than those reported for other donkey breeds such as dwarf Indian grey (21), Nordestina (38) or Martina Franca (63). Phosphorus is a mineral that contributes to bone health, together with calcium, magnesium and vitamin D. It is therefore valuable to calculate the calcium/phosphorus ratio. The calcium/phosphorus ratio in this study was 1.30, close to the one found in the work of Fantuz (63). Milk intended for children is recommended to meet the calcium/phosphorus ratio (1–2:1), thus donkey milk would be suitable for this group (22).

The sodium content was much lower than the reported levels in other species such as the semi-arid Brazilian Nordestina (38) and the dwarf Indian grey breed (21). This significant fact may be due to multiple factors such as the breed itself and also the diet these animals receive. The feeding of the animals participating in the study was based on pasture, organic alfalfa and oat supplementation. The low sodium concentration of the Zamorano-Leonese breed is of great importance as a functional feed. Cardiovascular diseases are one of the main causes of mortality in developed countries and there is a strong correlation between sodium intake and the development of hypertension (64). Compared to other species, it has lower levels than goat’s, sheep’s or cow’s milk (36) but similar to human milk (22).

Milk from Zamorano-Leonese donkeys had intermediate levels between Nordestina (38) and Martina Franca (63). A high level of potassium is recommended to prevent the development of hypertension (30).

The magnesium concentration is in line with that found in other breeds (38, 61). Human milk has a lower amount of magnesium than donkey milk (22). In contrast, cow’s, goat’s and sheep’s milk have 1.43, 1.90, and 2.14 times more magnesium than donkey’s milk (36).

The microelements analysed in donkey milk were zinc, iron, copper and selenium. The level of zinc found was slightly lower than that determined in Nordestina (38) and the dwarf Indian grey breed (21). However, the level of zinc is higher than that found in breast milk. This fact is relevant, as the requirements for this mineral have increased in childhood and adolescence, therefore these groups are at a higher risk of zinc deficiency. This deficiency could lead to stunted growth. Furthermore, the role of this mineral in the proper functioning of the immune system has been widely demonstrated.

Iron in Zamorano-Leonese donkey milk was lower than in the dwarf Indian grey breed (21). Human milk contains twice as much iron as donkey milk. However, the concentration of iron in donkey milk is in line with the content of the most widely consumed milks: cow’s, sheep’s or goat’s milk (36).

Regarding copper, this is the first time that this microelement has been measured in donkey milk, so we cannot compare it with other items. Zamorano-Leonese donkey milk contains twice as much copper as human milk and cow’s milk. These differences are even greater with other milks such as sheep’s or goat’s milk (36). Only mare’s milk has a higher concentration (56).

Selenium has also not been previously characterised in donkey milk. This mineral is involved in the enhancement of the immune system and in the protection of cells against oxidative damage. The amount of selenium in donkey milk is remarkable (10.71 μg/100 g) constituting 19.47% of the RDI. It contains more than ten times as much selenium as sheep’s and cow’s milk, and seven times more selenium than human milk (36).

## Conclusion

Zamorano-Leonese donkey milk did not show significant differences in physico-chemical properties compared to other donkey milk breeds. This milk is a highly nutrient-dense food, with a greater amount of protein than other donkey breeds. This species native to Spain has a low caloric and fat value, similar to skimmed cow’s milk.

Regarding the fatty acid profile, this donkey breed showed a higher amount of unsaturated fatty acids and a lower amount of saturated fatty acids than the others. Its major fatty acid is oleic fatty acid. Therefore, it has a more heart-healthy fat profile than other donkey breeds and other animal species. In terms of amino acid and protein profile there are no differences with other donkey breeds. Donkey milk has a high content of essential amino acids and α-lactoalbumin, lysozyme and lactoferrin. Its low concentration in β-lactoglobulin can be correlated to lower allergenicity.

Zamorano-Leonese donkey milk was notable for its high concentration of vitamin C and folic acid compared to other breeds and other milks. In relation to liposoluble vitamins, their content is lower due to the lower amount of fat, with the exception of vitamin D. In this group, the Zamorano-Leonese breed showed more vitamin E than the other donkey breeds. The fat-soluble vitamins were affected by the pasteurisation treatment, in contrast to the water-soluble vitamins and minerals.

Zamorano-Leonese donkey milk has a higher calcium content than human milk and the calcium/phosphorus ratio is the suitable for proper calcium absorption. In addition, this breed presented a lower sodium content. Regarding microelements, its zinc and selenium concentrations are noteworthy.

In summary, donkey milk can be considered a functional food or ingredient with a heart-healthy fatty acid profile and a high concentration of antioxidant vitamins, such as vitamin C. It also contributes to the maintenance of a healthy immune system due to its naturally high concentration of folic acid and selenium.

This food can be incorporated into regular diets as a dairy product or as an ingredient for the formulation of new matrices, since it is rich in protein, sustainably produced and its composition supports these affirmations.

## Data Availability Statement

The original contributions presented in the study are included in the article/supplementary material, further inquiries can be directed to the corresponding author.

## Author Contributions

MC-A and IA: conceptualisation and supervision. MC-A, IA, J-MJ, and ML: methodology. IA, MC-A, J-MJ, ML, MC, and AC: investigation and writing and original draft preparation. All authors have read and agreed to the published version of the manuscript.

## Conflict of Interest

The authors declare that the research was conducted in the absence of any commercial or financial relationships that could be construed as a potential conflict of interest.

## Publisher’s Note

All claims expressed in this article are solely those of the authors and do not necessarily represent those of their affiliated organizations, or those of the publisher, the editors and the reviewers. Any product that may be evaluated in this article, or claim that may be made by its manufacturer, is not guaranteed or endorsed by the publisher.
